# The burden of bladder cancer and kidney cancer attributable to smoking in Latin America and the Caribbean from 1990 to 2023 and projections to 2050

**DOI:** 10.18332/tid/226570

**Published:** 2026-09-08

**Authors:** Jia Deng, Bing Yang, Zhenzhen Wu, Hao Wu, Huazheng Sun, Zhenming Xie, Bo Dai, Dongxin Tang

**Affiliations:** 1 Guizhou University of Traditional Chinese Medicine, Guiyang, China; 2 The First Affiliated Hospital of Guizhou University of Traditional Chinese Medicine, Guiyang, China; 3 Department of Urology, Key Laboratory of Organ Regeneration and Transplantation of Ministry of Education, and National-Local Joint Engineering Laboratory of Animal Models for Human Diseases, The First Hospital of Jilin University, Changchun, China; 4 The Affiliated Hospital of Zunyi Medical University, Zunyi, China

**Keywords:** bladder cancer, kidney cancer, smoking, global burden of disease, Latin America and the Caribbean

## Abstract

**INTRODUCTION:**

Tobacco smoking is a major modifiable risk factor for bladder and kidney cancer. In Latin America and the Caribbean (LAC), comprehensive longitudinal evidence on the smoking-attributable burden of these cancers remains limited. This study assessed the burden, temporal trends, socioeconomic inequalities, and future projections of smoking-attributable bladder and kidney cancer in LAC.

**METHODS:**

Data were obtained from the Global Burden of Disease (GBD) 2023 study to estimate deaths, disability-adjusted life years (DALYs), age-standardized mortality rate (ASMR), and age-standardized DALYs rate (ASDR) for smoking-attributable bladder and kidney cancer across 33 countries and territories in Latin America and the Caribbean from 1990 to 2023. Temporal trends were evaluated using joinpoint regression and average annual percent change (AAPC). Socioeconomic inequalities were assessed in relation to the sociodemographic index (SDI) using the slope index of inequality (SII) and concentration index (CIX). Future burden to 2050 was projected using a Bayesian age-period-cohort model.

**RESULTS:**

In 2023, smoking-attributable bladder cancer caused an estimated 1742 deaths and 35903 DALYs in LAC, while kidney cancer caused 637 deaths and 14947 DALYs. From 1990 to 2023, ASMR and ASDR for both cancers showed overall declining trends, although substantial regional heterogeneity was observed, with the Caribbean showing increasing burden. At the national level, the burden of both cancers was positively associated with SDI in 2023. Projections to 2050 indicate that age-standardized rates will continue to decline, whereas the absolute numbers of deaths and DALYs will increase.

**CONCLUSIONS:**

Age-standardized rates declined overall, whereas the absolute numbers of deaths and DALYs are projected to increase in Latin America and the Caribbean. Stronger tobacco control and better-prepared health systems are needed to address the growing cancer burden in aging populations.

## INTRODUCTION

Bladder and kidney cancers are major contributors to the global cancer burden, significantly affecting incidence and mortality rates on a worldwide scale^1^. As two major urological malignancies, bladder and kidney cancers impose a considerable public health burden worldwide, with part of this burden attributable to modifiable risk factors^2^.

Tobacco smoking stands as the single most significant and modifiable risk factor for both bladder and kidney cancer^2,3^. It is estimated that smoking is responsible for approximately 50% of bladder cancer (BC) cases and a considerable portion of kidney cancer (KC) cases^4,5^. The carcinogenic compounds in tobacco smoke are absorbed into the bloodstream, filtered by the kidneys, and concentrated in the urine. This prolonged exposure of the urothelium to these carcinogens initiates and promotes the molecular changes that lead to malignancy in the kidney and bladder^6^.

The Latin America and Caribbean (LAC) region provides a unique context for studying the impact of smoking on cancer burden. The region is currently navigating a complex demographic and epidemiological transition, characterized by rapid population aging and an increasing prevalence of non-communicable diseases^7-9^. In recent decades, numerous countries in the LAC region have achieved significant progress in enforcing tobacco control measures, resulting in a widespread reduction in smoking rates^10,11^. However, this progress is heterogeneous, and the region still faces challenges from high smoking rates in some populations and persistent interference from the tobacco industry^12^.

Despite the established link between smoking and these cancers, there is a scarcity of comprehensive studies that systematically evaluate the long-term burden specifically attributable to smoking in the LAC region. Understanding the trends, identifying disparities among different populations, and forecasting the future burden are critical for evidence-based policymaking and the strategic allocation of healthcare resources^13^. Such an analysis is essential to tailor public health interventions and prepare health systems for the evolving challenge posed by these preventable malignancies. Therefore, the study seeks to thoroughly evaluate the impact of smoking on bladder and kidney cancer in Latin America and the Caribbean from 1990 to 2050. Specifically, we seek to: 1) quantify the mortality and disability-adjusted life years (DALYs) in 2023; 2) analyze the temporal trends and patterns from 1990 to 2023; 3) evaluate the socioeconomic inequalities associated with this burden; and 4) project the potential future burden up to the year 2050.

## METHODS

### Data source and definitions

This study was a secondary analysis of aggregated data from the Global Burden of Disease Study 2023 (GBD 2023)^13^. We extracted annual data on deaths, disability-adjusted life years (DALYs), age-standardized mortality rate (ASMR), and age-standardized DALYs rate (ASDR) for bladder cancer and kidney cancer attributable to smoking. The analysis covered 33 countries and territories grouped into four GBD subregions. Andean Latin America included Bolivia (Plurinational State of), Ecuador, and Peru; the Caribbean included Antigua and Barbuda, Bahamas, Barbados, Belize, Bermuda, Cuba, Dominica, the Dominican Republic, Grenada, Guyana, Haiti, Jamaica, Puerto Rico, Saint Kitts and Nevis, Saint Lucia, Saint Vincent and the Grenadines, Suriname, Trinidad and Tobago, and the United States Virgin Islands; Central Latin America included Colombia, Costa Rica, El Salvador, Guatemala, Honduras, Mexico, Nicaragua, Panama, and Venezuela (Bolivarian Republic of); and Tropical Latin America included Brazil and Paraguay. From 1990 to 2023, data were amassed and divided by sex and 5-year age segments (from 30–34 to ≥95 years)^14^. The sociodemographic index (SDI)^13^, a composite measure of lag-distributed income per capita, average years of schooling, and total fertility rate, was used to assess socioeconomic inequalities.

### Trend analysis

To investigate the time trends of ASMR and ASDR for bladder and kidney cancer linked to smoking from 1990 to 2023, we applied joinpoint regression analysis. This method finds points in time where a major alteration in the trend occurs and assesses the trend’s magnitude within each segment found. The primary output of this analysis is the average annual percent change (AAPC), which provides a single summary measure of the trend over the entire period. A positive AAPC indicates a rising trend, while a negative value signifies a declining trend. The analysis was conducted for the overall region, each subregion, and individual countries and territories, as well as for different age groups. We conducted this analysis using the National Cancer Institute’s Joinpoint Regression Program (Version 4.9.1.0).

### Health inequality analysis

Health inequality was assessed from both a socioeconomic and a temporal perspective. First, we began by examining the connection between disease burden and socioeconomic development through the SDI. The relationships between the ASMR and ASDR of each cancer and the SDI values for all included countries and territories in 2023 were assessed using Pearson correlation analysis. Second, we quantified socioeconomic inequality using two standard metrics: the slope index of inequality (SII) and the concentration index (CIX). The SII is a regression-based measure that represents the absolute difference in a health outcome (e.g. ASDR) between the most and least advantaged groups on the socioeconomic scale (SDI). A positive SII reflects a greater burden on high-SDI groups. The CIX measures relative inequality and is derived from a concentration curve; its value ranges from -1 to +1. A positive CIX suggests that high-SDI populations bear a greater health burden, whereas a negative CIX suggests the burden is heavier on low-SDI populations.

### Forecasting future burden

We used a Bayesian age-period-cohort (BAPC) model to forecast the future burden of bladder and kidney cancer attributable to smoking until 2050. This statistical model is well-suited for analyzing and forecasting mortality and incidence rates by disentangling the effects of age, calendar period (time), and birth cohort. The model was used to project future ASMR, ASDR, and the absolute numbers of deaths and DALYs for the Latin America and Caribbean region as a whole. The projections incorporated 95% uncertainty intervals (UIs) to illustrate the range of feasible future values. The BAPC package in R software was used for all forecasting analyses.

### Statistical analysis

All analyses were performed separately for smoking-attributable bladder cancer and kidney cancer. Deaths, disability-adjusted life years (DALYs), age-standardized mortality rate (ASMR), and age-standardized DALYs rate (ASDR) were summarized by year, sex, age group, country, and GBD subregion. Age-standardized rates were expressed per 100000 population, and GBD estimates were presented with 95% uncertainty intervals (UIs).

Temporal trends in ASMR and ASDR from 1990 to 2023 were examined using joinpoint regression. Average annual percent changes (AAPCs) and their 95% confidence intervals (CIs) were estimated for the overall Latin America and Caribbean region, the four GBD subregions, individual countries and territories, and age groups. Trends were classified as increasing or decreasing when the corresponding 95% CI was entirely above or below zero, respectively; otherwise, they were considered stable.

Pearson correlation analysis was used to assess the associations of the sociodemographic index (SDI) with ASMR and ASDR across the included countries and territories in 2023. Socioeconomic inequalities were further evaluated using the slope index of inequality (SII) and the concentration index (CIX), representing absolute and relative inequality, respectively. A Bayesian age-period-cohort model was used to project ASMR, ASDR, deaths, and DALYs through 2050.

Statistical analyses were conducted using R software version 4.3.2. Joinpoint analyses were performed using the National Cancer Institute Joinpoint Regression Program version 4.9.1.0, and projections were generated using the BAPC package in R. A p<0.05 was considered statistically significant.

## RESULTS

### The burden in 2023

In 2023, smoking-attributable bladder cancer in Latin America and the Caribbean accounted for an estimated 1741.67 deaths (95% UI: 1409.52–2087.17) and 35902.64 DALYs (95% UI: 29054.38–42876.49), with an ASMR of 0.62 (95% UI: 0.50–0.74) and an ASDR of 12.10 (95% UI: 9.82–14.45) (Table 1). Smoking-attributable kidney cancer accounted for an estimated 636.85 deaths (95% UI: 375.29–990.49) and 14946.94 disability-adjusted life years (95% UI: 8870.38–22818.02), with an ASMR of 0.22 (95% UI: 0.13–0.34) and an ASDR of 4.86 (95% UI: 2.89–7.45).

**Table 1:** The burden of bladder cancer and kidney cancer attributable to smoking in Latin America and the Caribbean in 2023

*Location*	*Bladder cancer*				*Kidney cancer*			
	*Deaths*		*DALYs*		*Deaths*		*DALYs*	
	*Count* *(95% UI)*	*ASMR* *(95% UI)*	*Count* *(95% UI)*	*ASDR* *(95% UI)*	*Count* *(95% UI)*	*ASMR* *(95% UI)*	*Count* *(95% UI)*	*ASDR* *(95% UI)*
**Latin America and Caribbean**	1741.67 (1409.52–2087.17)	0.62 (0.5–0.74)	35902.64 (29054.38–42876.49)	12.1 (9.82–14.45)	636.85 (375.29–990.49)	0.22 (0.13–0.34)	14946.94 (8870.38–22818.02)	4.86 (2.89–7.45)
**GBD regions**								
Andean Latin America	82.19 (56.44–117.13)	0.29 (0.2–0.42)	1631.55 (1133.28–2315.88)	5.5 (3.85–7.85)	32.78 (16.68–58.98)	0.11 (0.06–0.2)	710.25 (358.45–1233.75)	2.33 (1.18–4.06)
Caribbean	291.72 (225.01–381.83)	1.14 (0.88–1.49)	6153.03 (4847.18–7923.41)	23.38 (18.43–30.12)	65.86 (37.53–99.54)	0.25 (0.14–0.38)	1590.96 (901.98–2414.07)	5.93 (3.36–8.97)
Central Latin America	378.38 (291.65–477.77)	0.33 (0.25–0.42)	7908.22 (6156.26–9935.61)	6.54 (5.08–8.21)	193.95 (112.4–307.78)	0.16 (0.09–0.26)	4616.26 (2631.2–7184.5)	3.67 (2.1–5.73)
Tropical Latin America	989.37 (804.99–1181.85)	0.87 (0.71–1.05)	20209.84 (16200.61–24315.23)	16.9 (13.58–20.35)	344.26 (194.07–550.71)	0.29 (0.16–0.47)	8029.48 (4661.46–12553.07)	6.46 (3.76–10.15)
**Countries**								
Antigua and Barbuda	0.29 (0.19–0.39)	0.54 (0.36–0.74)	6.79 (4.55–9.39)	12.08 (8.1–16.53)	0.08 (0.04–0.15)	0.15 (0.07–0.27)	2.14 (1.1–3.84)	3.69 (1.89–6.69)
Bahamas	0.7 (0.47–1.04)	0.4 (0.27–0.58)	16.85 (11.41–24.84)	8.47 (5.7–12.49)	0.27 (0.13–0.5)	0.14 (0.07–0.26)	7.21 (3.72–13.17)	3.33 (1.69–6.08)
Barbados	1.37 (0.89–2.03)	0.55 (0.36–0.82)	29.51 (19.15–43.69)	11.78 (7.66–17.43)	0.56 (0.29–1)	0.22 (0.12–0.4)	13.15 (7.01–23.33)	5.34 (2.85–9.46)
Belize	0.75 (0.5–1.05)	0.54 (0.36–0.75)	17.42 (11.86–24.4)	11.34 (7.67–15.89)	0.25 (0.13–0.41)	0.16 (0.08–0.27)	6.44 (3.37–10.61)	3.94 (2.05–6.51)
Bermuda	0.97 (0.67–1.33)	1.5 (1.03–2.06)	19.96 (13.49–27.2)	31.14 (20.98–42.35)	0.16 (0.08–0.27)	0.24 (0.13–0.42)	3.54 (1.92–6.13)	5.64 (3.05–9.73)
Bolivia (Plurinational State of)	19.43 (10.37–34.53)	0.51 (0.27–0.91)	444.48 (238.16–796.82)	10.42 (5.51–18.7)	4.93 (2.32–8.52)	0.12 (0.05–0.2)	121.87 (58.2–208.91)	2.67 (1.27–4.6)
Brazil	966.76 (784.36–1159.19)	0.87 (0.7–1.04)	19747.98 (15720.04–23752.89)	16.86 (13.5–20.22)	334.92 (184.28–532.76)	0.29 (0.16–0.46)	7802.51 (4490.37–12105.3)	6.42 (3.66–10)
Colombia	57.04 (41.26–77.64)	0.22 (0.16–0.3)	1246.4 (910.96–1658.29)	4.68 (3.45–6.26)	25.24 (12.93–42.94)	0.1 (0.05–0.16)	610.36 (320.04–999.56)	2.25 (1.18–3.72)
Costa Rica	15.73 (11.41–20.68)	0.58 (0.43–0.77)	317.58 (232.09–411.12)	11.08 (8.09–14.38)	5.87 (2.8–10.06)	0.21 (0.1–0.35)	134.7 (65.58–224.72)	4.53 (2.19–7.57)
Cuba	199.25 (149.79–266.85)	2.12 (1.59–2.83)	4141.41 (3162.34–5491.28)	44.11 (33.7–58.27)	43.49 (24.62–66.78)	0.46 (0.26–0.71)	1057.69 (609.35–1633.51)	11.27 (6.52–17.36)
Dominica	0.33 (0.2–0.51)	0.68 (0.42–1.04)	7.16 (4.46–10.75)	14.34 (8.96–21.59)	0.11 (0.04–0.2)	0.21 (0.09–0.4)	2.63 (1.13–4.96)	5.26 (2.27–9.86)
Dominican Republic	19.25 (11.56–31.71)	0.45 (0.26–0.75)	408.38 (255.89–654.24)	8.93 (5.51–14.4)	3.51 (1.49–6.81)	0.08 (0.03–0.15)	82.61 (35.94–152.88)	1.73 (0.75–3.25)
Ecuador	21.4 (15.16–28.71)	0.26 (0.19–0.35)	424.35 (304.09–562.45)	5.1 (3.65–6.77)	9.07 (4.82–15.35)	0.11 (0.06–0.18)	201.22 (110.48–338.31)	2.38 (1.31–4.01)
El Salvador	5.04 (3.07–7.87)	0.21 (0.13–0.33)	106.47 (64.87–160.72)	4.28 (2.61–6.46)	1.75 (0.69–3.42)	0.07 (0.03–0.14)	41.02 (16.85–80.23)	1.64 (0.67–3.2)
Grenada	0.31 (0.19–0.44)	0.39 (0.24–0.56)	7.15 (4.56–10.1)	8.89 (5.64–12.62)	0.1 (0.05–0.19)	0.12 (0.07–0.23)	2.48 (1.37–4.51)	3.05 (1.68–5.56)
Guatemala	6.73 (4.47–9.86)	0.14 (0.09–0.21)	140.45 (92.71–205.36)	2.74 (1.8–4.02)	4.38 (2.32–7.89)	0.09 (0.04–0.16)	102.67 (54.8–183.78)	1.91 (1.02–3.42)
Guyana	1.86 (1.31–2.69)	0.56 (0.39–0.79)	48.5 (34.8–68.67)	13.34 (9.55–19.01)	0.52 (0.27–0.93)	0.15 (0.08–0.26)	14.85 (7.74–25.77)	3.9 (2.02–6.83)
Haiti	9.16 (4.23–15.33)	0.3 (0.14–0.51)	233.8 (110.41–386.81)	6.66 (3.1–11.18)	1.44 (0.61–2.82)	0.04 (0.02–0.09)	39.02 (16.77–74.96)	1.05 (0.45–2.05)
Honduras	3.48 (1.84–6.18)	0.12 (0.06–0.22)	65.31 (35.08–115.79)	2.08 (1.11–3.7)	1.47 (0.66–2.84)	0.05 (0.02–0.09)	32.87 (15.37–62.35)	0.99 (0.46–1.88)
Jamaica	9.83 (6.59–13.6)	0.66 (0.44–0.91)	223.88 (154.63–302.44)	14.61 (10.06–19.77)	2.02 (1.02–3.63)	0.13 (0.07–0.24)	49.7 (26.25–88.31)	3.21 (1.69–5.71)
Mexico	199.93 (150.51–261.08)	0.34 (0.25–0.44)	4118.91 (3115.08–5420.91)	6.61 (4.98–8.68)	128.62 (69.37–202.85)	0.21 (0.11–0.32)	3046.87 (1668.57–4792.86)	4.71 (2.57–7.4)
Nicaragua	3.46 (1.88–5.73)	0.18 (0.1–0.31)	80.12 (43.64–126.59)	3.7 (2.03–6)	2.42 (1.08–4.44)	0.11 (0.05–0.21)	61.05 (27.76–112.27)	2.65 (1.2–4.87)
Panama	3.48 (2.23–4.9)	0.17 (0.11–0.23)	69.45 (44.3–101.18)	3.22 (2.06–4.68)	2.68 (1.41–4.78)	0.12 (0.07–0.22)	62.18 (32.45–106.29)	2.83 (1.48–4.86)
Paraguay	22.61 (15.45–32.58)	1.02 (0.69–1.47)	461.86 (320.9–666.45)	18.94 (13.09–27.11)	9.34 (4.14–15.97)	0.38 (0.16–0.65)	226.96 (102.17–386.02)	8.5 (3.8–14.58)
Peru	41.36 (27.84–58.73)	0.26 (0.17–0.37)	762.72 (517.53–1094.44)	4.49 (3.03–6.38)	18.79 (8.58–36.29)	0.11 (0.05–0.22)	387.16 (176.57–734.77)	2.21 (1.01–4.19)
Puerto Rico	27.41 (20.42–35.23)	0.67 (0.5–0.86)	539.05 (408.76–678.46)	14.26 (10.88–17.92)	8.11 (4.22–13.47)	0.21 (0.11–0.35)	178.16 (94.09–290.66)	5.14 (2.69–8.35)
Saint Kitts and Nevis	0.12 (0.08–0.17)	0.47 (0.31–0.7)	2.62 (1.69–3.82)	9.54 (6.26–14.06)	0.03 (0.02–0.06)	0.13 (0.06–0.24)	0.85 (0.41–1.58)	2.91 (1.39–5.39)
Saint Lucia	0.82 (0.56–1.17)	0.69 (0.47–0.98)	19.29 (13.17–27.69)	15.82 (10.8–22.68)	0.16 (0.08–0.28)	0.13 (0.07–0.23)	4.27 (2.21–7.27)	3.44 (1.78–5.87)
Saint Vincent and the Grenadines	0.36 (0.23–0.52)	0.43 (0.28–0.62)	8.37 (5.41–12.03)	10.04 (6.49–14.38)	0.06 (0.03–0.11)	0.07 (0.03–0.13)	1.55 (0.77–2.77)	1.87 (0.93–3.33)
Suriname	2.02 (1.22–2.99)	0.7 (0.42–1.04)	48.02 (28.77–71.83)	15.43 (9.26–23.16)	0.61 (0.29–1.06)	0.2 (0.09–0.34)	16.23 (7.74–28.91)	4.94 (2.34–8.65)
Trinidad and Tobago	7.02 (5.02–9.77)	0.81 (0.57–1.14)	165.74 (120.44–230.2)	17.43 (12.66–24.35)	2.02 (1.08–3.31)	0.22 (0.11–0.36)	51.61 (28.14–82.67)	5.28 (2.86–8.48)
United States Virgin Islands	0.27 (0.18–0.35)	0.28 (0.19–0.38)	5.63 (3.84–7.45)	6.35 (4.42–8.4)	0.18 (0.09–0.3)	0.2 (0.1–0.34)	4.22 (2.25–7.05)	5.15 (2.82–8.5)
Venezuela (Bolivarian Republic of)	83.48 (57–117.62)	0.71 (0.49–1)	1763.53 (1199.12–2514.25)	13.09 (8.86–18.55)	21.52 (11.17–36.93)	0.16 (0.08–0.27)	524.54 (268.28–899.38)	3.52 (1.82–6.03)

DALYs: disability-adjusted life years. ASMR: age-standardized mortality rate per 100000 population. ASDR: age-standardized DALYs rate per 100000 population.

Among the four GBD subregions, the Caribbean exhibited the highest ASDR for BC at 23.38 (95% UI: 18.43–30.12), followed by Tropical Latin America (16.9; 95% UI: 13.58–20.35). Tropical Latin America recorded the highest absolute number of BC deaths (989.37; 95% UI: 804.99–1181.85) and DALYs (20209.84; 95% UI: 16200.61–24315.23). For KC, Tropical Latin America also demonstrated the highest ASDR (6.46; 95% UI: 3.76–10.15) and the largest number of DALYs (8029.48; 95% UI: 4661.46–12553.07). At the national level, Cuba had a particularly high burden, with an ASDR of 44.11 for BC and 11.27 for KC. Brazil contributed the largest absolute burden, with 966.76 BC deaths (95% UI: 784.36–1159.19) and 334.92 KC deaths (95% UI: 184.28–532.76) (Table 1).

**Figure 1:**
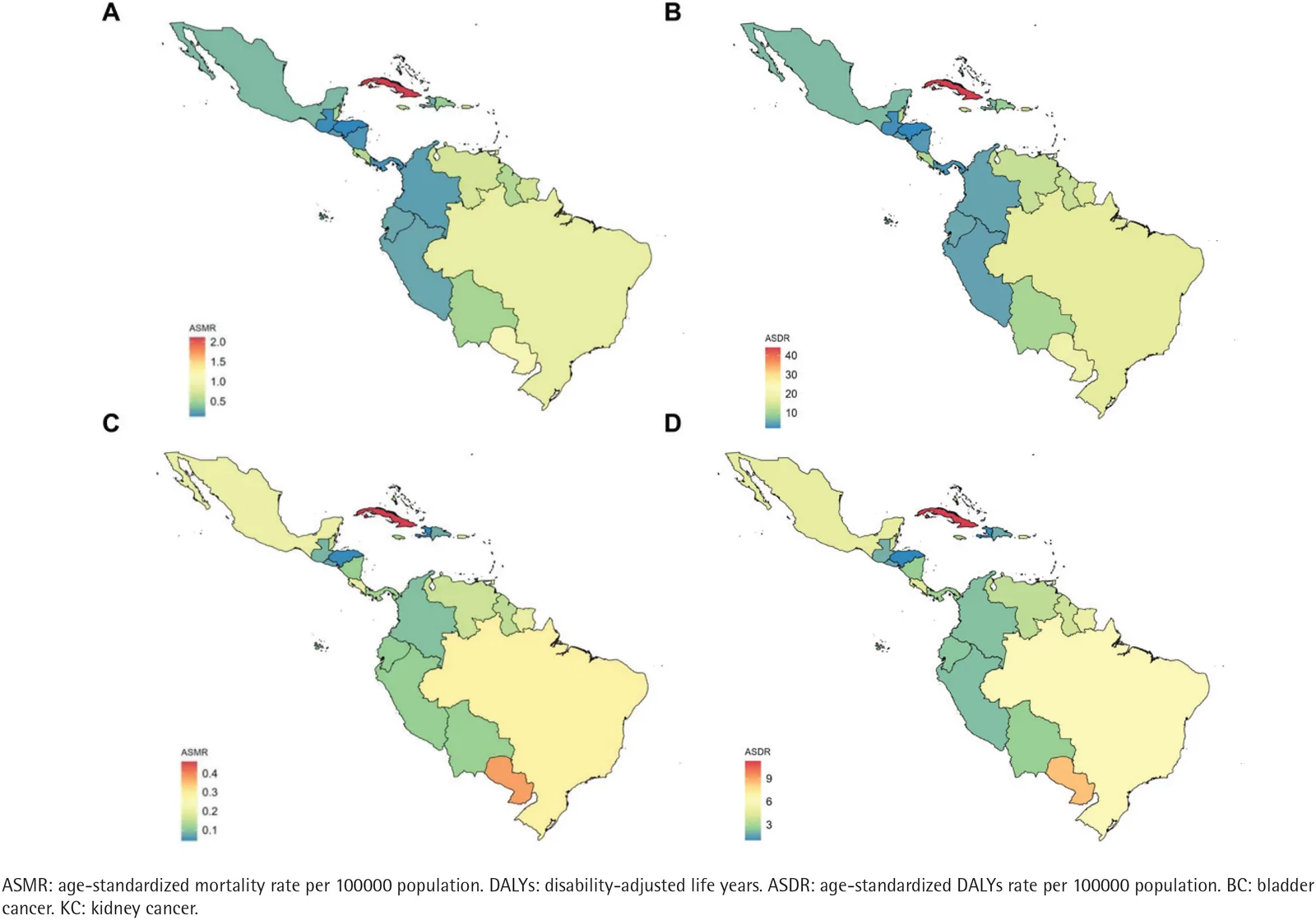
The burden of BC and KC attributable to smoking in Latin America and the Caribbean among males in 2023: A) ASMR of BC; B) ASDR of BC; C) ASMR of KC; D) ASDR of KC

The burden of both cancers increased with age. For BC, ASDR rose from 0.33 in the 30–34 age group to a peak of 127.8 in the 85–89 age group (95% UI: 97.93–169.66), while the ASMR was highest among those aged ≥95 years (11.83; 95% UI: 7.89–17.23). Similarly, for KC, the highest ASDR was observed in the age group 75–79 years (33.88; 95% UI: 18.7–54.09), and the peak ASMR occurred in the age group of 80–84 years (2.35; 95% UI: 1.25–4.01) (Figure 1 and Table 2).

**Table 2:** The burden of bladder cancer and kidney cancer attributable to smoking, by age, in Latin America and the Caribbean in 2023

*Age (years)*	*Bladder cancer*				*Kidney cancer*			
	*Deaths*		*DALYs*		*Deaths*		*DALYs*	
	*Count* *(95% UI)*	*ASMR* *(95% UI)*	*Count* *(95% UI)*	*ASDR* *(95% UI)*	*Count* *(95% UI)*	*ASMR* *(95% UI)*	*Count* *(95% UI)*	*ASDR* *(95% UI)*
30–34	1.15 (0.91–1.47)	0.01 (0–0.01)	72.6 (56.58–93.6)	0.33 (0.26–0.42)	0.33 (0.14–0.54)	0 (0–0)	19.57 (8.36–32.12)	0.09 (0.04–0.15)
35–39	2.55 (1.97–3.28)	0.01 (0.01–0.02)	145.07 (113.22–185.87)	0.68 (0.53–0.87)	1.19 (0.62–1.89)	0.01 (0–0.01)	65.42 (33.75–104.48)	0.31 (0.16–0.49)
40–44	6.52 (5.01–8.27)	0.03 (0.02–0.04)	330.04 (254.07–420.75)	1.62 (1.25–2.06)	3.99 (2.25–6.26)	0.02 (0.01–0.03)	197 (110–307.12)	0.97 (0.54–1.51)
45–49	16.74 (13.09–20.34)	0.09 (0.07–0.11)	757.34 (600.34–935.18)	4.22 (3.34–5.21)	11.47 (6.72–17.08)	0.06 (0.04–0.1)	507.56 (295.01–756.14)	2.83 (1.64–4.21)
50–54	39.91 (31.97–48.73)	0.25 (0.2–0.3)	1609.78 (1272.43–1961.85)	9.99 (7.89–12.17)	29.41 (17.38–42.93)	0.18 (0.11–0.27)	1154.18 (678.78–1682.47)	7.16 (4.21–10.44)
55–59	91.91 (74.86–109.71)	0.64 (0.52–0.76)	3248.02 (2632.22–3855.13)	22.64 (18.35–26.88)	62.43 (37.5–93.07)	0.44 (0.26–0.65)	2144.98 (1287.7–3208.37)	14.95 (8.98–22.37)
60–64	174.04 (139.86–206.61)	1.45 (1.16–1.72)	5291.15 (4245.91–6272.46)	44.01 (35.31–52.17)	94 (55.03–141.92)	0.78 (0.46–1.18)	2789.37 (1625.79–4209.3)	23.2 (13.52–35.01)
65–69	259.79 (208.7–316.38)	2.77 (2.22–3.37)	6653.39 (5344.29–8055.15)	70.85 (56.91–85.78)	119.01 (69.72–180.6)	1.27 (0.74–1.92)	2974.82 (1746.9–4503.63)	31.68 (18.6–47.96)
70–74	317.01 (250.81–387.1)	4.6 (3.64–5.62)	6649.07 (5264.34–8169.14)	96.53 (76.42–118.59)	110.62 (62.94–174.07)	1.61 (0.91–2.53)	2275.43 (1291.4–3591.38)	33.03 (18.75–52.14)
75–79	323.57 (256.79–398.53)	7.06 (5.6–8.7)	5404.1 (4298.44–6609.2)	117.95 (93.81–144.25)	94.46 (52.47–151.41)	2.06 (1.15–3.3)	1552.41 (856.76–2478.25)	33.88 (18.7–54.09)
80–84	251.31 (196.59–313.75)	9.13 (7.14–11.39)	3250.43 (2543.54–4048.38)	118.05 (92.37–147.03)	64.66 (34.48–110.34)	2.35 (1.25–4.01)	827.46 (440.94–1412.95)	30.05 (16.01–51.31)
85–89	170.98 (130.78–227.2)	12.48 (9.55–16.59)	1750.54 (1341.33–2323.87)	127.8 (97.93–169.66)	32.28 (16.39–56.74)	2.36 (1.2–4.14)	327.01 (165.61–573.8)	23.87 (12.09–41.89)
90–94	63.92 (46.88–88.78)	12.49 (9.16–17.34)	563.84 (413.13–782.85)	110.14 (80.7–152.92)	10.12 (4.69–19.34)	1.98 (0.92–3.78)	88.69 (41.18–168.64)	17.32 (8.04–32.94)
≥95	22.26 (14.84–32.43)	11.83 (7.89–17.23)	177.28 (118.21–257.76)	94.2 (62.81–136.96)	2.88 (1.26–5.63)	1.53 (0.67–2.99)	23.03 (10.02–44.88)	12.24 (5.32–23.85)

DALYs: disability-adjusted life years. ASMR: age-standardized mortality rate per 100000 population. ASDR: age-standardized DALYs rate per 100000 population.

### Trends from 1990 to 2023

From 1990 to 2023, the total impact of bladder and kidney cancer linked to smoking decreased in Latin America and the Caribbean. The ASMR and ASDR for both types of cancer decreased with an AAPC of -0.47% (95% CI: -0.55 – -0.39) and -0.70% (95% CI: -0.79 – -0.59), respectively. However, these trends varied across the subregions. Central Latin America experienced the most significant decline, with an AAPC of -1.09% for both ASMR and ASDR. In contrast, the Caribbean showed a significant increasing trend, with an AAPC of 0.54% for ASMR and 0.41% for ASDR. Andean Latin America demonstrated a slight increase in ASMR (AAPC=0.11%) but a slight decrease in ASDR (AAPC= -0.04%) (Supplementary file: Figures S1–S4 and Table 2).

At the national level, there was considerable heterogeneity in the trends. Cuba recorded the most substantial increase in the burden for both cancers, with an AAPC for ASMR of 1.47% (95% CI: 0.98–1.77). Other countries like Bolivia (Plurinational State of) and Belize also showed increasing trends. Conversely, Bermuda experienced the steepest decline, with an AAPC for ASMR of -2.28% (95% CI: -2.65 – -1.91). Significant decreasing trends were also observed in several other nations, including Venezuela (Bolivarian Republic of), the Dominican Republic, and Panama (Supplementary file: Figure 2D and Table 2).

**Figure 2:**
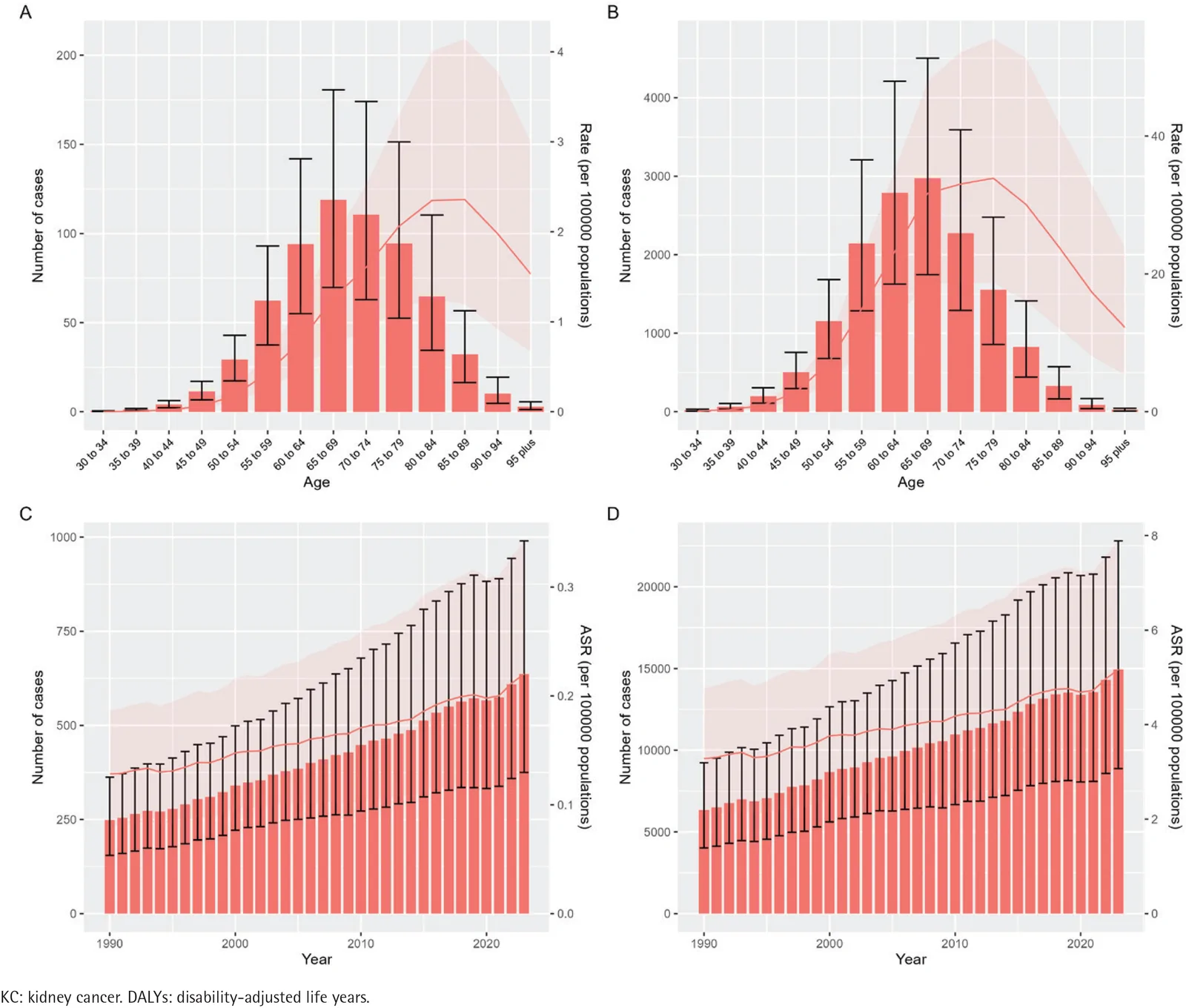
The burden of KC attributable to smoking in Latin America and the Caribbean among males: A) Death by age group; B) DALYs by age group; C) Death by year; D) DALYs by year

Analysis by age group revealed a distinct pattern for both bladder and kidney cancer. A significant decreasing trend in both ASMR and ASDR was observed in the younger to middle-aged populations (aged 30–74 years). The rate of decline was most pronounced in the age group 30–39 years, with an AAPC for ASMR around -2.3%. However, this trend reversed in the oldest age groups. For individuals aged ≥85 years, the burden showed a significant increasing trend. The most notable increase was in the age group 90–94 years, with an AAPC for ASMR of 1.19% (95% CI: 1.01–1.34).

### Health inequality

**Figure 3:**
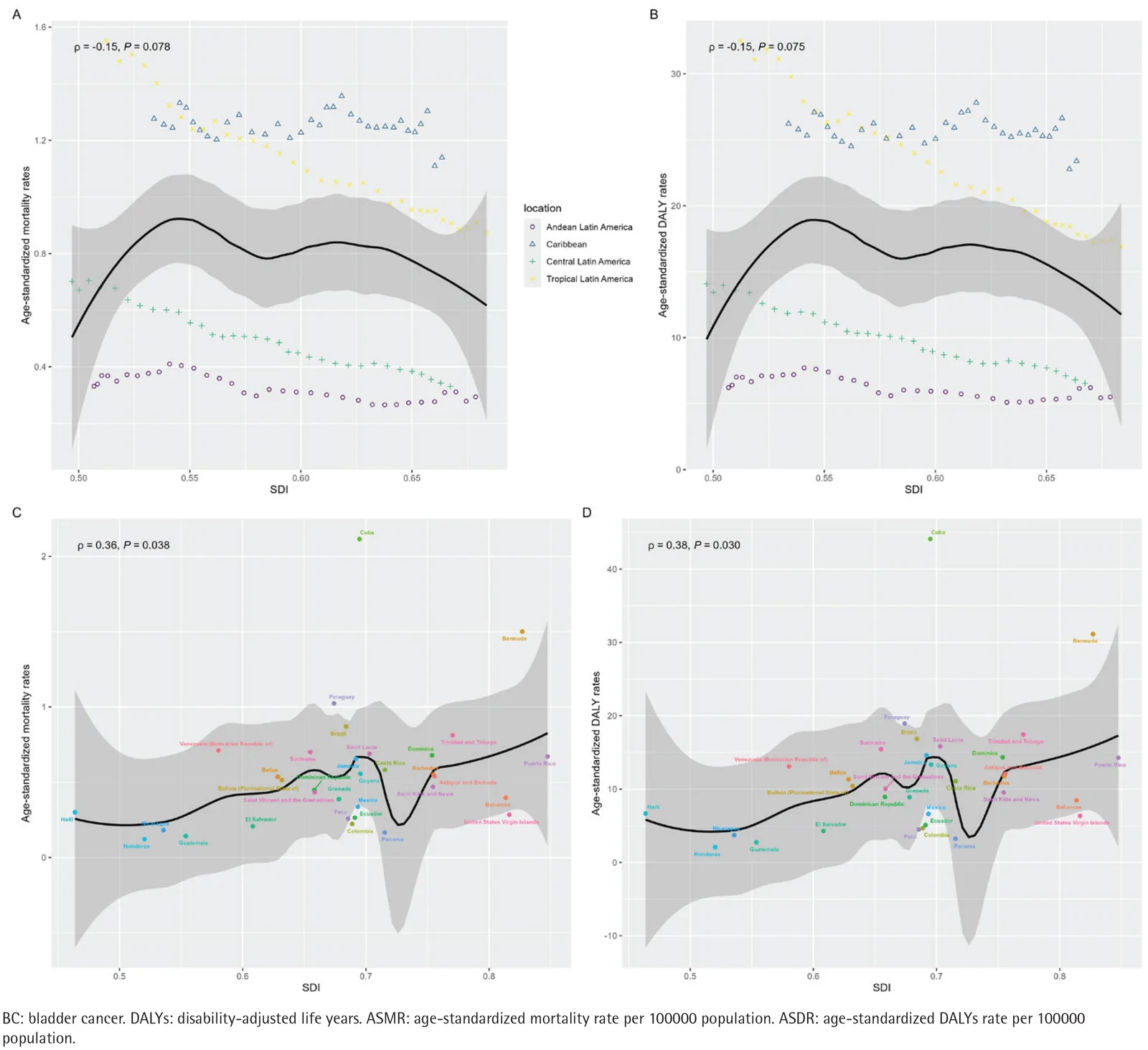
Correlation analysis of BC attributable to smoking among regions and countries in Latin America and the Caribbean between 1990 and 2023: A) ASMR among regions; B) ASDR among regions; C) ASMR among countries; D) ASDR among countries

**Figure 4:**
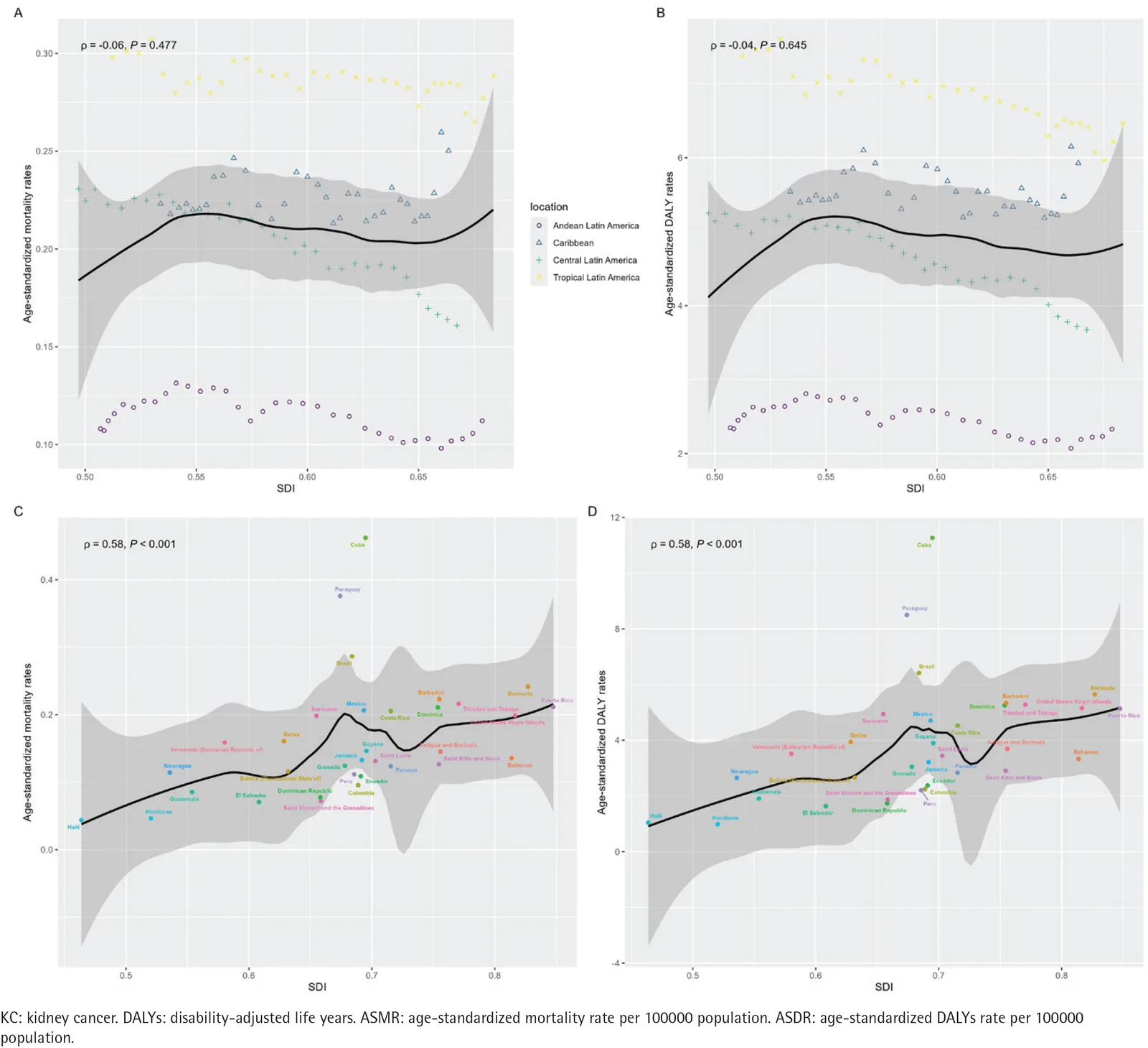
Correlation analysis of KC attributable to smoking among regions and countries in Latin America and the Caribbean between 1990 and 2023. A) ASMR among regions; B) ASDR among regions; C) ASMR among countries; D) ASDR among countries

The burden of smoking-attributable bladder and kidney cancer in Latin America and the Caribbean showed a significant positive correlation with the SDI at the national level in 2023. For BC, the ASMR and ASDR both had a significant positive association with SDI (r=0.36, p=0.038 for deaths; r=0.38, p=0.030 for DALYs). An even stronger positive correlation was observed for KC, where the ASMR and ASDR were highly correlated with SDI (r=0.58, p<0.001 for both). However, at the regional level, no significant correlation was found between the burden of either cancer and SDI (Figures 3 and 4).

Absolute inequality, as measured by the slope index of inequality (SII), indicated that the burden of smoking-attributable BC was disproportionately concentrated in countries with higher SDI. In 1990, the SII for deaths was 0.34 (p=0.014) and for DALYs was 6.96 (p=0.012). While this inequality persisted in 2023 with a significant positive SII for both deaths (0.17; p=0.035) and DALYs (4.11; p=0.022), the magnitude of the index decreased over the period, suggesting a reduction in absolute inequality. For KC, the SII was not statistically significant in either 1990 or 2023 (Supplementary file Figures S3 and S8).

Relative inequality, assessed by the concentration index (CIX), showed a shift over time for BC. In 1990, the CIX for deaths (0.019) and DALYs (0.012) was positive but not statistically significant, indicating a slight concentration of the burden in higher-SDI countries. By 2023, the CIX became negative for both deaths (-0.055) and DALYs (-0.044), suggesting a shift in the relative burden towards lower SDI countries, although these results were not statistically significant. For KC, a significant positive CIX was observed in 1990 for both deaths (0.080, p=0.013) and DALYs (0.069, p=0.042), indicating that the burden was concentrated in countries with higher SDI. This relative inequality diminished by 2023, with the CIX for both measures becoming non-significant (Supplementary file Figures S3 and S8).

### ASR BAPC prediction

Predictions for the impact of smoking on bladder and kidney cancer in Latin America and the Caribbean by 2050 show a steady drop in age-standardized rates, but an increase in the overall number of cases. For BC, the ASMR is predicted to decrease from 0.56 (95% UI: 0.53–0.59) in 2024 to 0.29 (95% UI: 0.01–0.56) in 2050. Similarly, the ASDR is projected to fall from 11.14 (95% UI: 10.71–11.57) to 5.01 (95% UI: -3.99–14.02) over the same period. Despite these declining rates, the absolute number of deaths is expected to rise from 1847 in 2024 to 2470 in 2050, and the number of DALYs is projected to increase from 36931 to 51633 (Supplementary file Figure S5).

For KC, a similar trend is anticipated. The ASMR is projected to decrease from 0.20 (95% UI: 0.18–0.21) in 2024 to 0.12 (95% UI: -0.01–0.25) in 2050. The ASDR is forecasted to show a slight increase from 4.69 (95% UI: 4.51–4.87) to 5.34 (95% UI: -3.12–13.80), although the wide uncertainty interval for the 2050 projection should be noted. In contrast to the rate trends, the absolute burden is expected to grow substantially. The number of deaths from smokingattributable KC is predicted to increase from 648 in 2024 to 999 in 2050, while the number of DALYs is projected to more than triple, rising from 15651 to 47191 (Supplementary file Figure S6).

## DISCUSSION

This study presents a comprehensive longitudinal assessment of the smoking-attributable burden of bladder and kidney cancer in LAC, highlighting a notable public health paradox: although ASMR and ASDR have declined, the absolute numbers of deaths and DALYs are projected to increase markedly by 2050. Our findings underscore the dual impact of successful tobacco control policies and the inexorable challenge of demographic transition, highlighting the complex landscape of cancer control in the region.

The observed decline in age-standardized rates is a testament to the public health victories achieved through tobacco control. Since the adoption of the WHO Framework Convention on Tobacco Control (FCTC), many LAC nations have made significant strides in implementing key policies, such as taxation, smoke-free environments, and warning labels^10,15,16^. This has contributed to a notable decrease in regional smoking prevalence, from 28% in 2000 to 16.3% in 2020^17,18^. The declining age-standardized rates for smoking-attributable cancers in our study are a direct reflection of these successes, consistent with global analyses showing that effective tobacco control can bend the curve of attributable disease^19,20^. The declining age-standardized rates observed in our study are consistent with broader evidence of progress in tobacco control across the region^19,20^. Age-specific analyses showed declining ASMR and ASDR among adults aged 30–74 years, with the most pronounced decline in ASMR among those aged 30–39 years, whereas increasing trends were observed among adults aged ≥85 years.

However, this progress is being counteracted by a powerful demographic tide. The LAC region is undergoing a rapid demographic transition, characterized by both sustained population growth and accelerated aging^21,22^. The number of individuals aged >65 years in the region is projected to more than double between 2024 and 2050^23^. As the incidence of bladder and kidney cancer rises sharply with age, this demographic shift creates an expanding population base at high risk, thus driving an increase in the absolute number of cases, deaths, and DALYs. Our projections align with global forecasts that also predict a rising absolute cancer burden, even in the face of declining age-standardized rates, primarily due to population growth and aging^24,25^. This paradox – falling rates but rising numbers – is the central challenge for future cancer control planning in the LAC region.

Our analysis of health inequalities reveals a nuanced and evolving picture. The positive correlation between the cancer burden and the SDI at the national level in 2023 suggests that more developed countries within the LAC region currently bear a greater burden. This is consistent with findings from other studies showing that the burden of smoking-related cancers has historically been concentrated in higher-SDI regions, likely due to a longer legacy of high smoking prevalence and potentially better cancer detection and registration^14,26,27.^ However, our finding that the absolute inequality (SII) for BC has decreased over time, while the relative inequality (CIX) shows a non-significant shift towards lower-SDI countries, may signal an epidemiological transition. As higher-SDI countries advance more rapidly in tobacco control and cancer care, the burden may become increasingly concentrated in less-resourced nations, a pattern observed in other non-communicable diseases^28^. This potential shift necessitates a proactive focus on strengthening cancer control infrastructure in lower-SDI settings to prevent the widening of health disparities.

The considerable heterogeneity in trends across the LAC region underscores that a one-size-fits-all approach to tobacco and cancer control is insufficient. The alarming increase in smokingattributable cancer burden in the Caribbean, for example, contrasts sharply with the steep declines in Central America. This divergence may be explained by variations in the timing and stringency of FCTC policy implementation, differences in historical and current smoking prevalence, and the varying intensity of tobacco industry interference, which remains a significant barrier to progress in some parts of the region^29,30.^ The unique trajectory of Cuba, with its high historical smoking rates, further highlights the long-lasting impact of tobacco use on national cancer profiles^31,32^.

### Strengths and limitations

This study has several notable strengths, including the use of comprehensive and standardized data from the GBD 2023 database, enabling a robust long-term evaluation of temporal trends, socioeconomic disparities, and future projections across a geographically and demographically diverse region. Nevertheless, some limitations must be acknowledged. First, GBD estimates are model-based and depend on the quality and availability of primary data from individual countries; limited data in certain LAC nations may therefore compromise the precision of the estimates. Second, as an ecological study, our findings describe population-level trends and cannot be used to infer individual-level risk. Finally, our analysis is specific to smoking attribution and does not account for other risk factors such as occupational exposures, obesity, or genetic predispositions, which also contribute to the burden of bladder and kidney cancer^33,34^.

## CONCLUSIONS

Age-standardized rates of mortality and disability-adjusted life years for smoking-attributable bladder and kidney cancer declined overall in Latin America and the Caribbean from 1990 to 2023, although patterns varied across subregions, countries and territories, and age groups. By 2050, the absolute numbers of deaths and disability-adjusted life years are projected to increase for both cancers. Projected age-standardized rates decline for bladder cancer and kidney cancer mortality, whereas the age-standardized disability-adjusted life years rate for kidney cancer shows a slight increase with wide uncertainty.

## Supplementary Material



## Data Availability

All data were obtained from the public open database: Global Health Data Exchange (GHDx) query tool (http://ghdx.healthdata.org/gbd-results-tool).
