# Supplementary material for: The burden of bladder cancer and kidney cancer attributable to smoking in Latin America and the Caribbean from 1990 to 2023 and projections to 2050
Source: Tob Induc Dis. 2026 Sep 8;24:10.18332/tid/226570. doi: 10.18332/tid/226570 (PMC13576406; doi:10.18332/tid/226570)
Supplement: Supplementary file 1 [file TID-24-159-s1.pdf]

Tobacco Induced Diseases

Supplementary file

© 2026 Deng J. et al.

DOI:
10.18332/tid/226570

The content has been provided by the author(s) and has not been reviewed, verified, or endorsed by European Publishing. It may not have undergone peer review. The views, opinions, and recommendations expressed are solely those of the author(s) and do not necessarily reflect the position of European Publishing. European Publishing accepts no responsibility or liability for any consequences arising from the use of, or reliance on, this content.

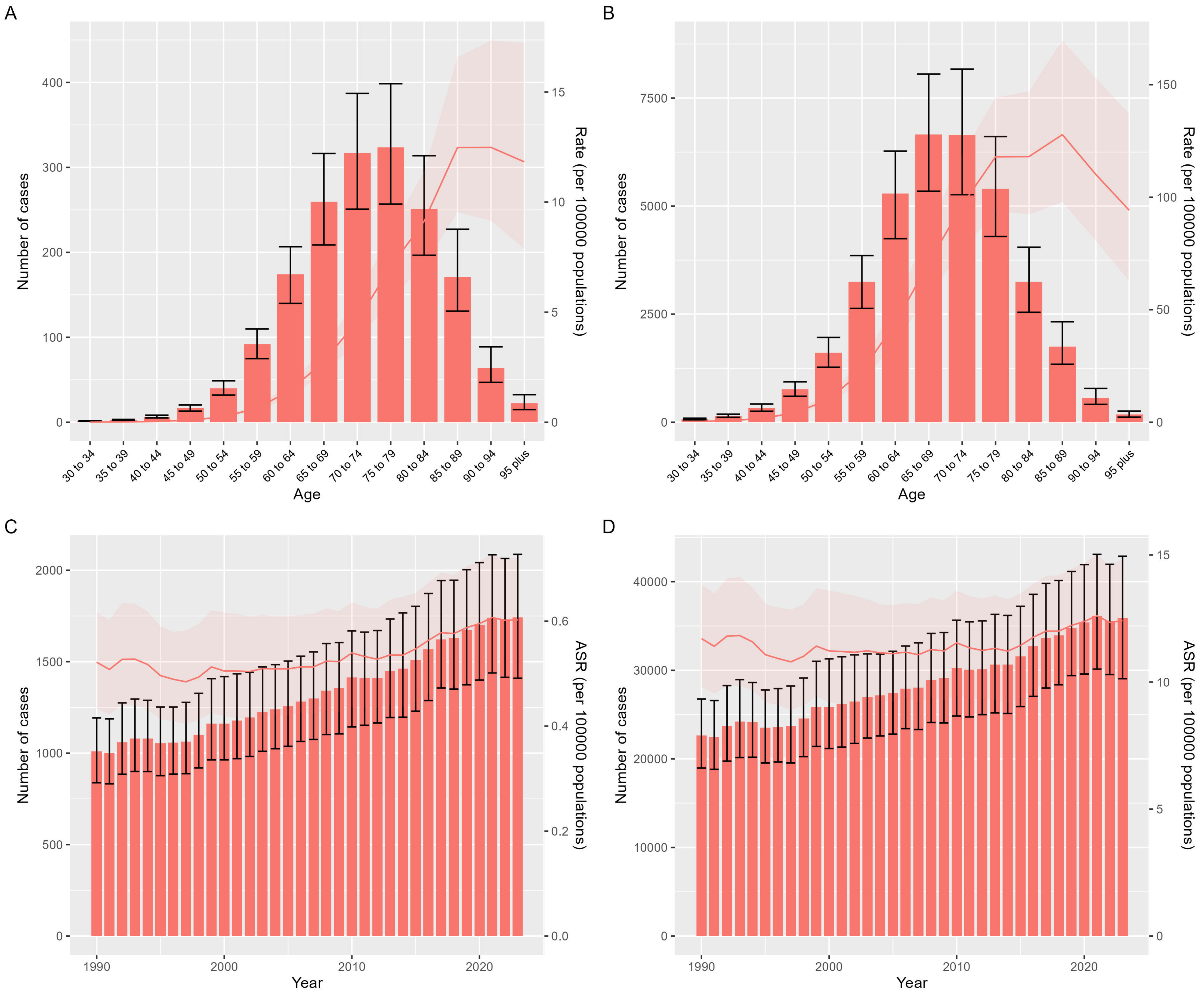

A
B
C
D
Number of cases
Rate (per 100000 populations)
ASR (per 100000 populations)
Age
Year
30 to 34
35 to 39
40 to 44
45 to 49
50 to 54
55 to 59
60 to 64
65 to 69
70 to 74
75 to 79
80 to 84
85 to 89
90 to 94
95 plus
1990
2000
2010
2020

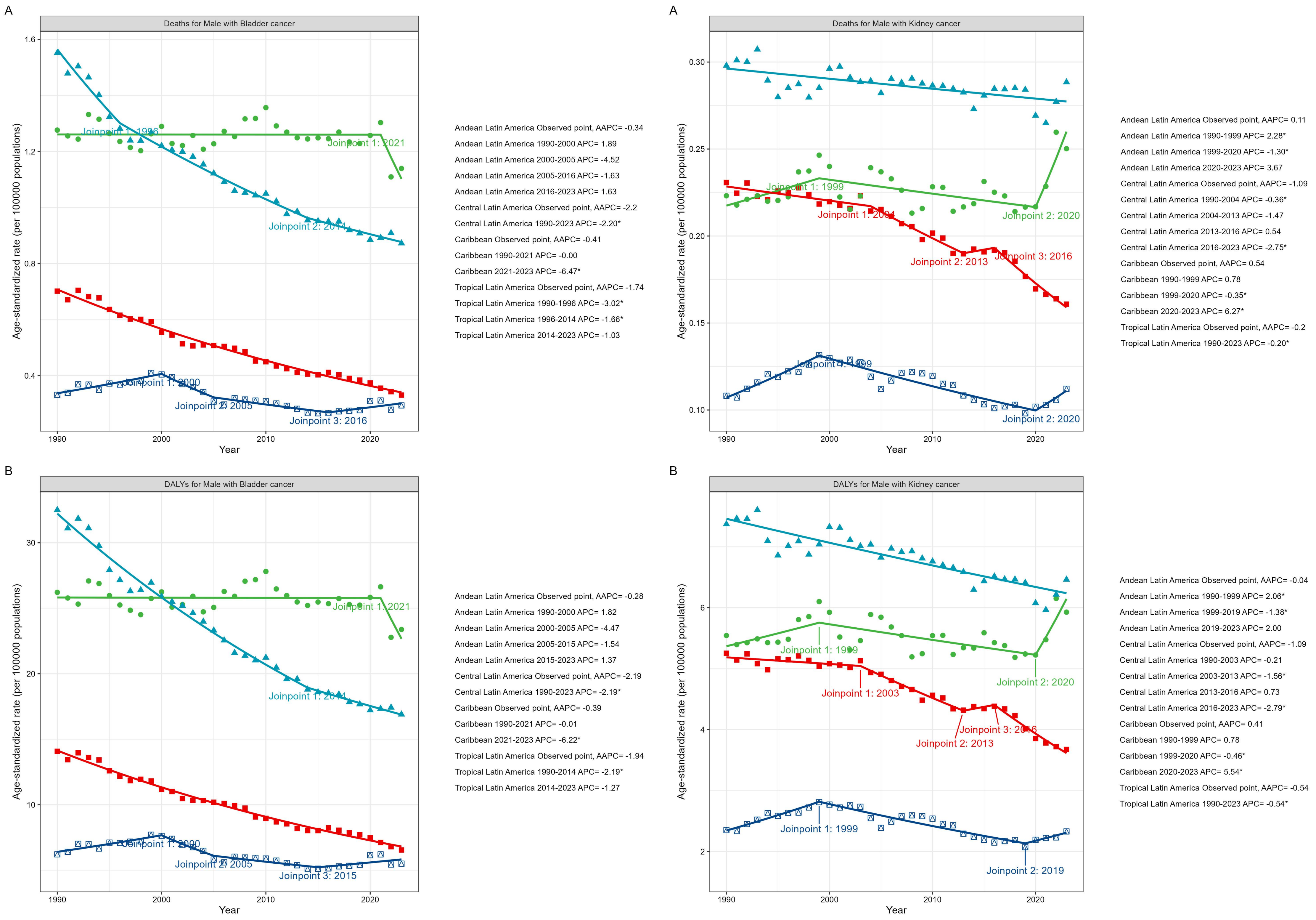
A
Deaths for Male with Bladder cancer
Age-standardized rate (per 100000 populations)
Year
Joinpoint 1: 1996
Joinpoint 1: 2021
Joinpoint 2: 2014
Joinpoint 1: 2000
Joinpoint 2: 2005
Joinpoint 3: 2016
Andean Latin America Observed point, AAPC= -0.34
Andean Latin America 1990-2000 APC= 1.89
Andean Latin America 2000-2005 APC= -4.52
Andean Latin America 2005-2016 APC= -1.63
Andean Latin America 2016-2023 APC= 1.63
Central Latin America Observed point, AAPC= -2.2
Central Latin America 1990-2023 APC= -2.20*
Caribbean Observed point, AAPC= -0.41
Caribbean 1990-2021 APC= -0.00
Caribbean 2021-2023 APC= -6.47*
Tropical Latin America Observed point, AAPC= -1.74
Tropical Latin America 1990-1996 APC= -3.02*
Tropical Latin America 1996-2014 APC= -1.66*
Tropical Latin America 2014-2023 APC= -1.03
A
Deaths for Male with Kidney cancer
Age-standardized rate (per 100000 populations)
Year
Joinpoint 1: 1999
Joinpoint 2: 2020
Joinpoint 1: 2004
Joinpoint 2: 2013
Joinpoint 3: 2016
Joinpoint 1: 1999
Joinpoint 2: 2020
Andean Latin America Observed point, AAPC= 0.11
Andean Latin America 1990-1999 APC= 2.28*
Andean Latin America 1999-2020 APC= -1.30*
Andean Latin America 2020-2023 APC= 3.67
Central Latin America Observed point, AAPC= -1.09
Central Latin America 1990-2004 APC= -0.36*
Central Latin America 2004-2013 APC= -1.47
Central Latin America 2013-2016 APC= 0.54
Central Latin America 2016-2023 APC= -2.75*
Caribbean Observed point, AAPC= 0.54
Caribbean 1990-1999 APC= 0.78
Caribbean 1999-2020 APC= -0.35*
Caribbean 2020-2023 APC= 6.27*
Tropical Latin America Observed point, AAPC= -0.2
Tropical Latin America 1990-2023 APC= -0.20*
B
DALYs for Male with Bladder cancer
Age-standardized rate (per 100000 populations)
Year
Joinpoint 1: 2021
Joinpoint 1: 2014
Joinpoint 1: 2000
Joinpoint 2: 2005
Joinpoint 3: 2015
Andean Latin America Observed point, AAPC= -0.28
Andean Latin America 1990-2000 APC= 1.82
Andean Latin America 2000-2005 APC= -4.47
Andean Latin America 2005-2015 APC= -1.54
Andean Latin America 2015-2023 APC= 1.37
Central Latin America Observed point, AAPC= -2.19
Central Latin America 1990-2023 APC= -2.19*
Caribbean Observed point, AAPC= -0.39
Caribbean 1990-2021 APC= -0.01
Caribbean 2021-2023 APC= -6.22*
Tropical Latin America Observed point, AAPC= -1.94
Tropical Latin America 1990-2014 APC= -2.19*
Tropical Latin America 2014-2023 APC= -1.27
B
DALYs for Male with Kidney cancer
Age-standardized rate (per 100000 populations)
Year
Joinpoint 1: 1999
Joinpoint 2: 2020
Joinpoint 1: 2003
Joinpoint 2: 2013
Joinpoint 3: 2016
Joinpoint 1: 1999
Joinpoint 2: 2019
Andean Latin America Observed point, AAPC= -0.04
Andean Latin America 1990-1999 APC= 2.06*
Andean Latin America 1999-2019 APC= -1.38*
Andean Latin America 2019-2023 APC= 2.00
Central Latin America Observed point, AAPC= -1.09
Central Latin America 1990-2003 APC= -0.21
Central Latin America 2003-2013 APC= -1.56*
Central Latin America 2013-2016 APC= 0.73
Central Latin America 2016-2023 APC= -2.79*
Caribbean Observed point, AAPC= 0.41
Caribbean 1990-1999 APC= 0.78
Caribbean 1999-2020 APC= -0.46*
Caribbean 2020-2023 APC= 5.54*
Tropical Latin America Observed point, AAPC= -0.54
Tropical Latin America 1990-2023 APC= -0.54*

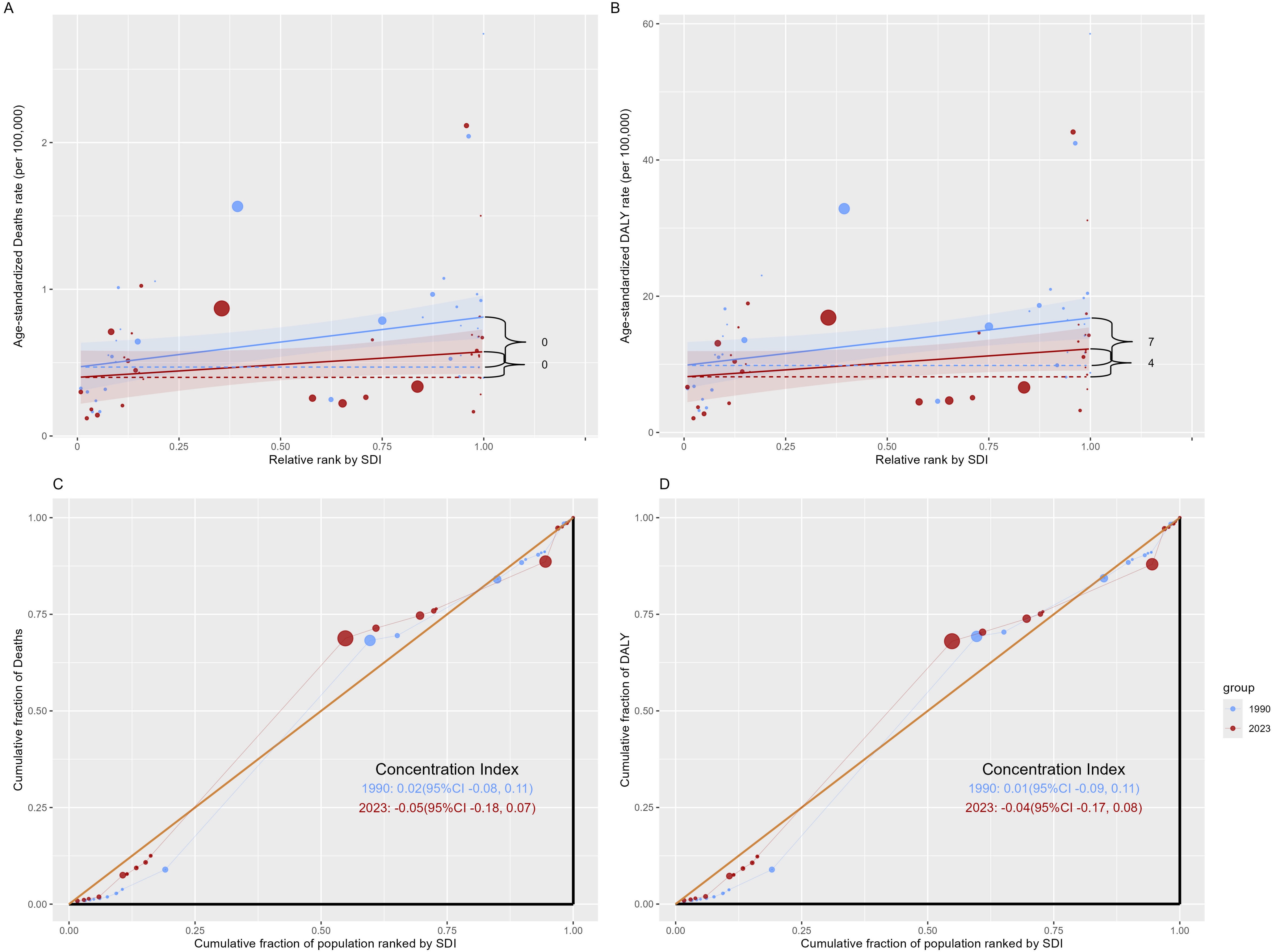

A
Age-standardized Deaths rate (per 100,000)
Relative rank by SDI
0
0
B
Age-standardized DALY rate (per 100,000)
Relative rank by SDI
7
4
C
Cumulative fraction of Deaths
Cumulative fraction of population ranked by SDI
Concentration Index
1990: 0.02(95%CI -0.08, 0.11)
2023: -0.05(95%CI -0.18, 0.07)
D
Cumulative fraction of DALY
Cumulative fraction of population ranked by SDI
Concentration Index
1990: 0.01(95%CI -0.09, 0.11)
2023: -0.04(95%CI -0.17, 0.08)
group
1990
2023

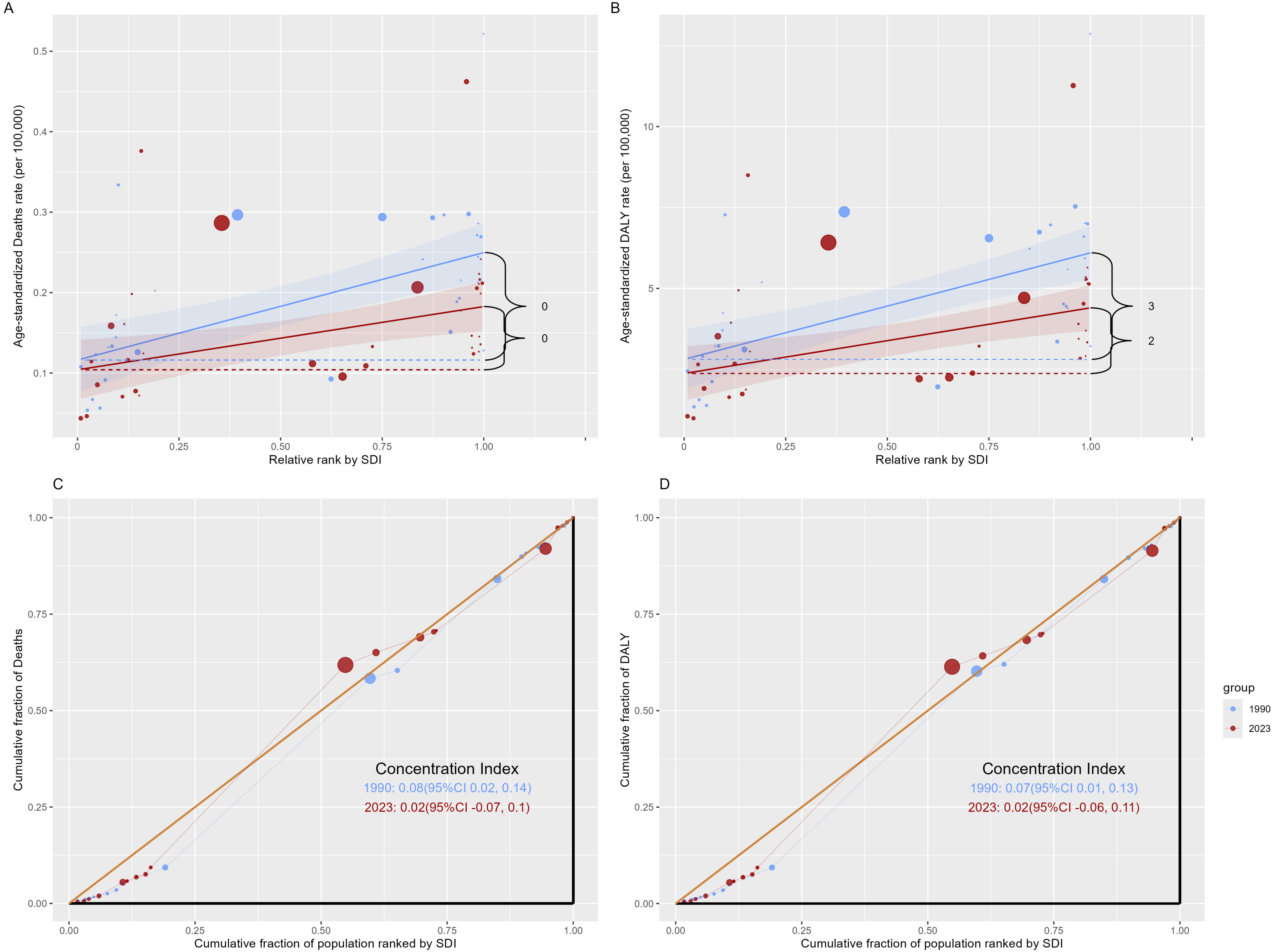

A
Age-standardized Deaths rate (per 100,000)
Relative rank by SDI
0
0
B
Age-standardized DALY rate (per 100,000)
Relative rank by SDI
3
2
C
Cumulative fraction of Deaths
Cumulative fraction of population ranked by SDI
Concentration Index
1990: 0.08(95%CI 0.02, 0.14)
2023: 0.02(95%CI -0.07, 0.1)
D
Cumulative fraction of DALY
Cumulative fraction of population ranked by SDI
Concentration Index
1990: 0.07(95%CI 0.01, 0.13)
2023: 0.02(95%CI -0.06, 0.11)
group
1990
2023

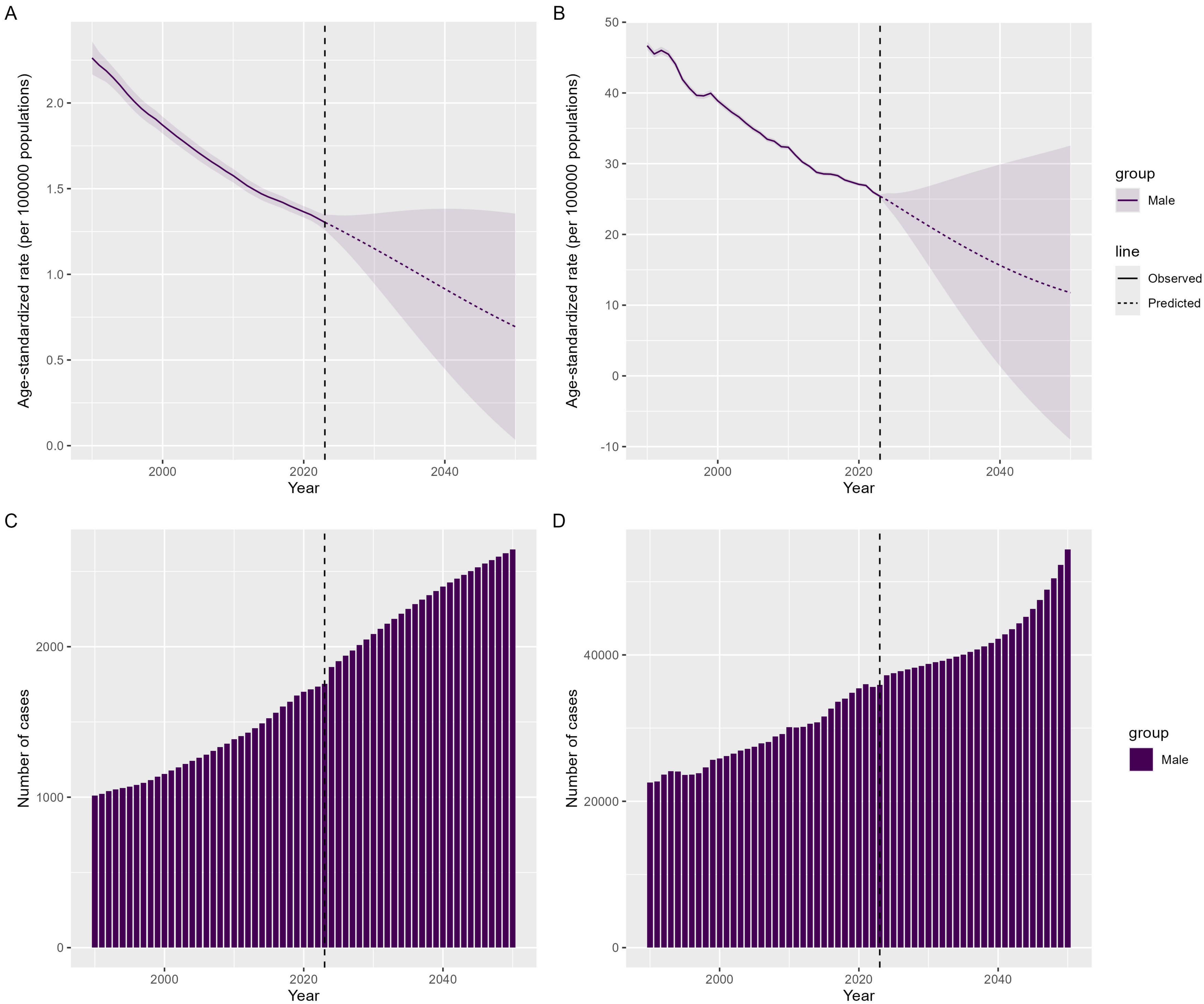
A
B
C
D
Age-standardized rate (per 100000 populations)
Number of cases
Year
2000
2020
2040
0.0
0.5
1.0
1.5
2.0
-10
0
10
20
30
40
50
1000
2000
20000
40000
group
Male
line
Observed
Predicted

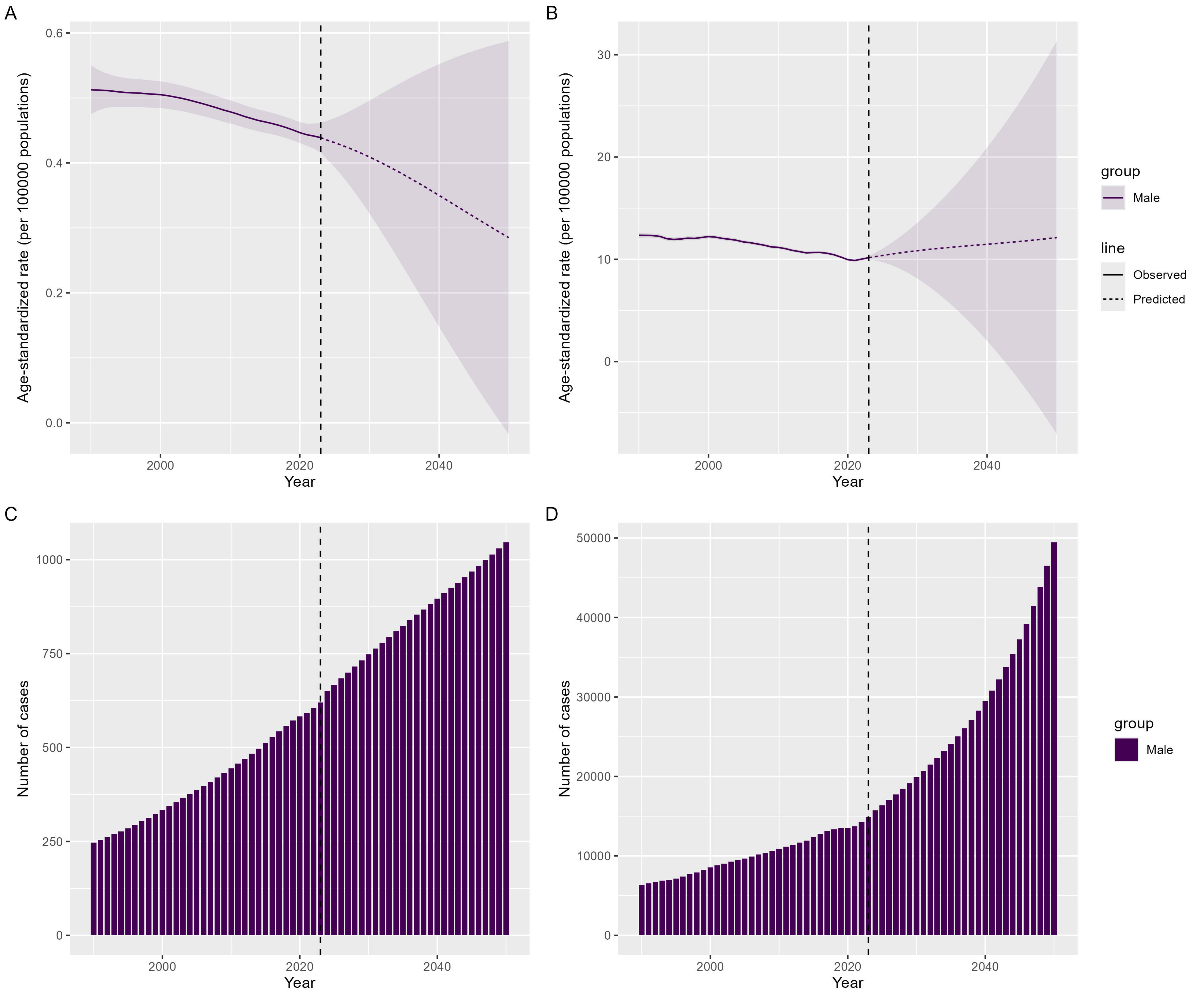
A
B
C
D
Age-standardized rate (per 100000 populations)
Number of cases
Year
0.0
0.2
0.4
0.6
0
10
20
30
250
500
750
1000
10000
20000
30000
40000
50000
2000
2020
2040
group
Male
line
Observed
Predicted
